# Metal Complexes of Bispidine Derivatives: Achievements and Prospects for the Future

**DOI:** 10.3390/molecules30051138

**Published:** 2025-03-03

**Authors:** Altynay B. Kaldybayeva, Valentina K. Yu, Feyyaz Durap, Murat Aydemir, Khaidar S. Tassibekov

**Affiliations:** 1 Faculty of Chemistry and Chemical Technology, Al Farabi Kazakh National University, 71 Al-Farabi Ave, Almaty 050040, Kazakhstan; kh.tassibekov@ihn.kz; 2 Laboratory of Chemistry of Synthetic and Natural Medicinal Substances, A.B. Bekturov Institute of Chemical Sciences, 106 Sh. Ualikhanov St., Almaty 050010, Kazakhstan; 3 Department of Chemistry, Faculty of Science, Dicle University, 21280 Diyarbakir, Türkiye; feyyazdurap@gmail.com (F.D.); aydemir@dicle.edu.tr (M.A.); 4 Science and Technolgy, Application and Research Center (DUBTAM), Dicle University, 21280 Diyarbakir, Türkiye

**Keywords:** bispidine, bispidine framework, metal complexes, synthesis, radiopharmaceuticals, catalyst

## Abstract

Multidentate bispidine ligands, including tetra-, penta-, hexa-, hepta-, and octadentate variants, exhibit strong coordination tendencies due to their intrinsic rigidity, significant reorganization potential, and ability to efficiently encapsulate metal ions. These structural attributes profoundly influence the thermodynamic stability, metal ion selectivity, redox behavior, and spin-state configuration of the resulting complexes. Metal ions, in turn, serve as highly suitable candidates for coordination due to their remarkable kinetic inertness, rapid complex formation kinetics, and low redox potential. This review focuses on ligands incorporating the bispidine core (3,7-diazabicyclo[3.3.1]nonane) and provides an overview of advancements in the synthesis of metal complexes involving p-, d-, and f-block elements. Furthermore, the rationale behind the growing interest in bispidine-based complexes for applications in radiopharmaceuticals, medicinal chemistry, and organic synthesis is explored, particularly in the context of their potential for diagnostic and catalytic drug development.

## 1. Introduction

**Figure 1:**
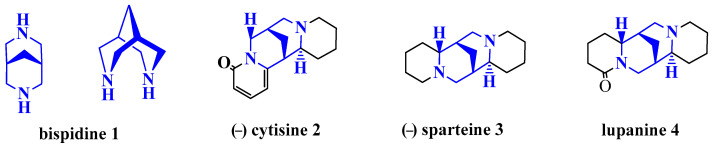
Bispidine framework and natural alkaloids containing it.

Bispidine 1 (3,7-diazabicyclo[3.3.1]nonane), composed of two condensed piperidine rings, was first synthesized by Mannich and Moss in the 1930s through the condensation of piperidine with paraformaldehyde and primary amines [1]. Its coordination with transition metals was later established by Stetter and Haller in 1957 and 1969, respectively [2]. Since then, the bispidine framework has been extensively studied in coordination chemistry [3,4,5], organic synthesis [6,7], radiopharmaceuticals [8,9,10,11], and medical chemistry [12]. Naturally occurring bispidine derivatives are found in *Genista* and *Lupinus* species in the form of the cyclic alkaloids such as cytisine 2, spartein 3, and lupanin 4 (Figure 1), which exhibit antiarrhythmic, anticonvulsant, and antimicrobial properties [13,14,15].

**Scheme 1:**
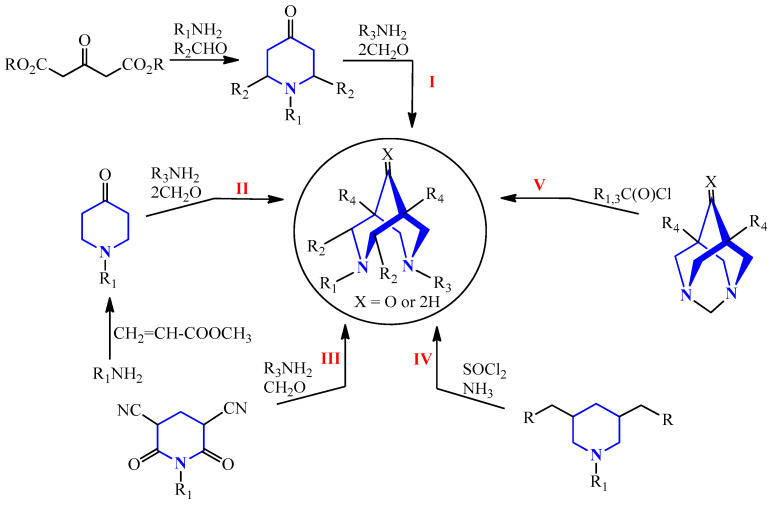
Synthesis methods of 3,7-diazabicyclo[3.3.1]nonanes. (I) Mannich reaction-based synthesis from carbonyl compounds and aliphatic amines [16]; (II) transformation of 4-piperidones into 3,7-diazabicyclo[3.3.1]nonanes [17]; (III) acid hydrolysis of carboxylic acid nitrile derivatives, reduction and, in some cases, alkylation (or acylation with subsequent reduction) of the obtained 3,7-diazabicyclo[3.3.1]nonanes [18]; (IV) cyclization of 3,5-bifunctional piperidine derivatives [13]; (V) ring-opening of the 1,3-diazaadamantane cycle [19].

Derivatives of 3,7-diazabicyclo[3.3.1]nonane can typically be synthesized through five distinct methods (Scheme 1):

## 2. The Structure of Bispidine Ligands

**Figure 2:**
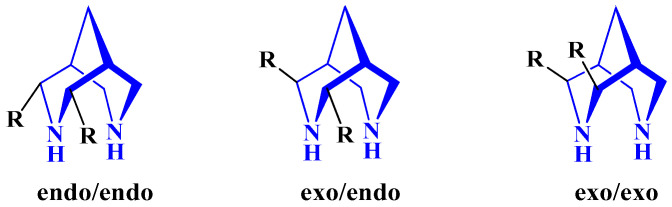
Possible configuration isomers of bispidine.

The bispidine frame can adopt three primary conformations: armchair–armchair, armchair–boat (or boat–chair), and boat–boat. Among these, the armchair–armchair conformation is generally the most energetically favorable and is particularly suitable for capturing one or two protons via intramolecular hydrogen bonding (N–H···N and N–H···Cl···H–N). This conformation also facilitates efficient metal chelation. The donor centers, specifically N7 and N3, contribute to the rigidity of the ligand framework, while the incorporation of additional donor groups, such as pyridyl moieties at C2 and C4, introduces structural flexibility. Furthermore, bispidine ligans can exhibit configurational isomerism (Figure 2), resulting in different ligand orientations: endo/endo ligands (fully equatorial), exo/endo (axial and equatorial), and exo/exo (fully axial). The endo/endo configuration is particularly advantageous for creating a well-organized coordination environment, ensuring optimal donor positioning for metal complexation [20,21,22].

**Figure 3:**
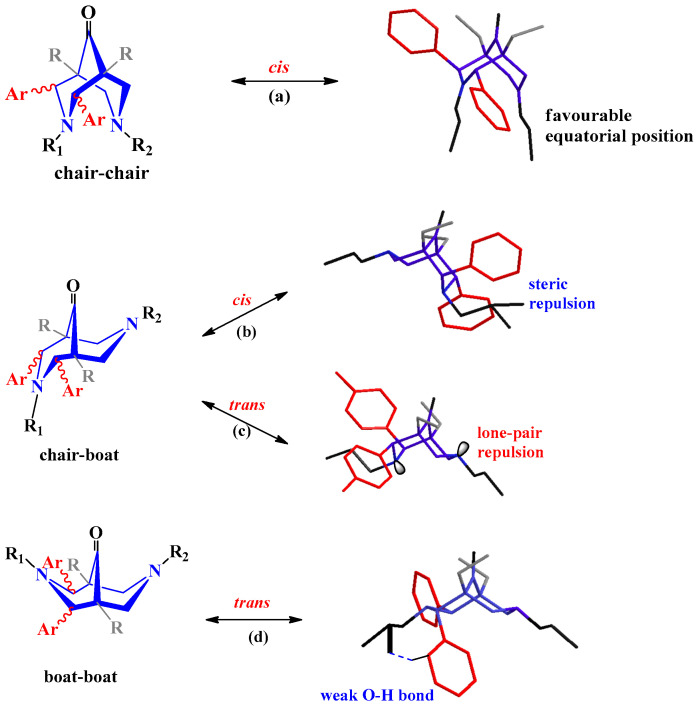
Structural conformations of bispidons. (**a**) cis-chair–chair, (**b**) cis-chair–boat, (**c**) trans-chair–boat, (**d**) trans-boat–chair.

Studies have demonstrated that NMR spectroscopy can effectively determine the cis/trans configurations (Figure 3) of substituents at the 2nd and 4th positions [23]. In the armchair–armchair conformation, phenyl substituents at C2 and C4 adopt equatorial positions, resulting in a cis-symmetric configuration. However, the introduction of bulky substituents or hydrogen bonds accepting groups at N3 induces a transition to the trans-configuration.

**Figure 4:**
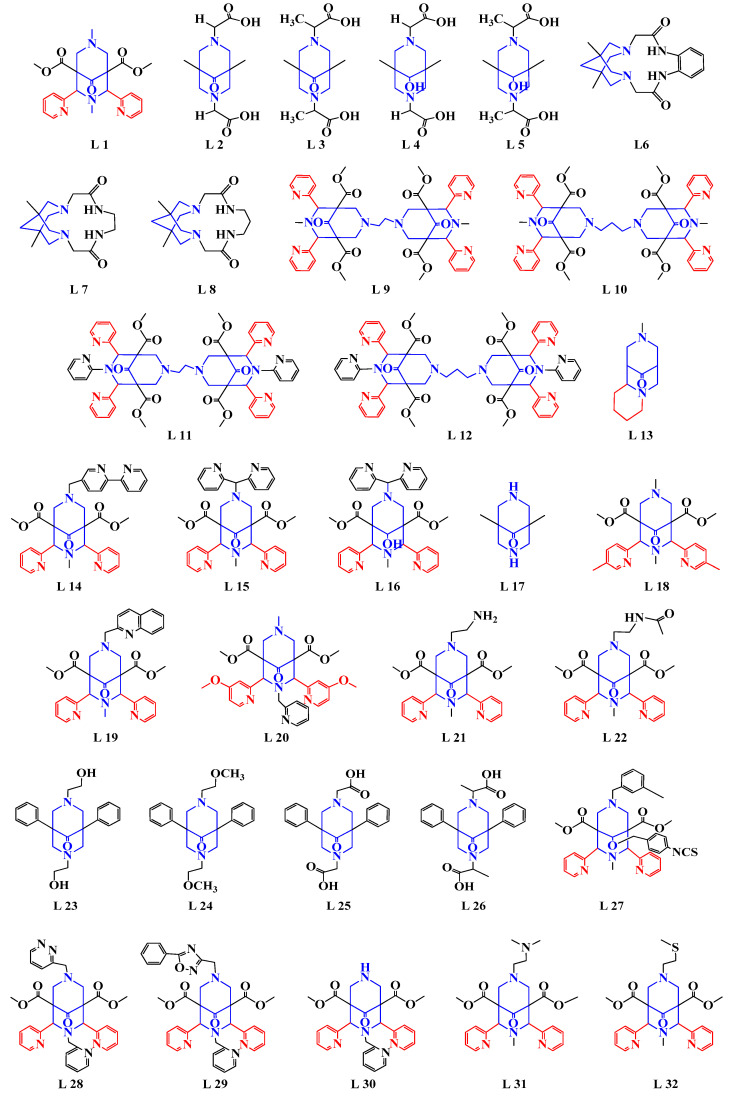

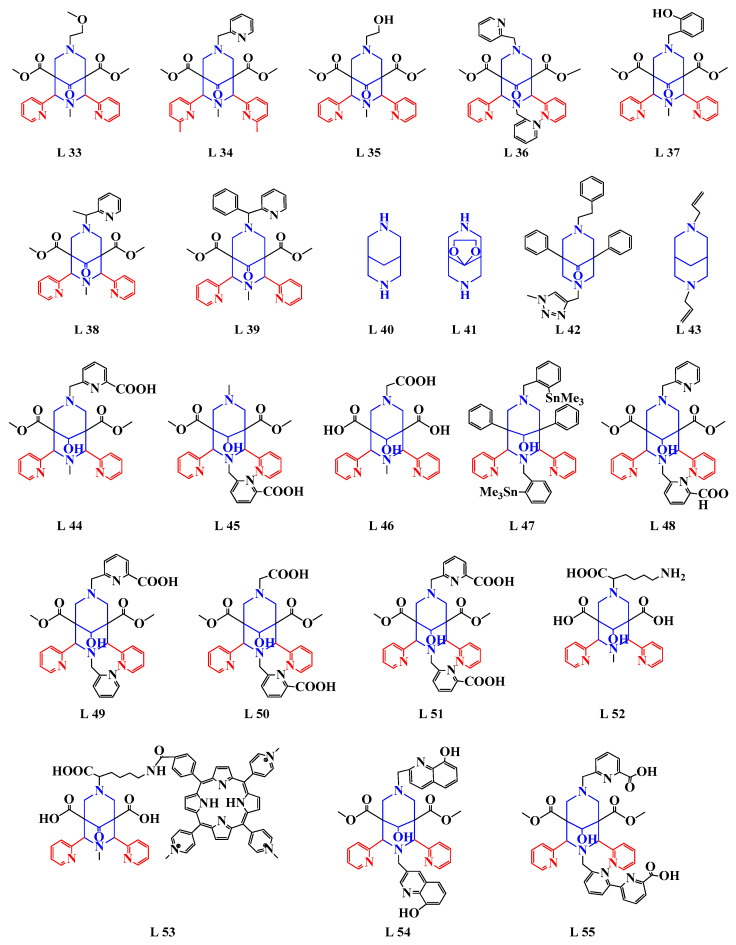
Structures of bispidine ligands.

The structures of all ligands and the characteristics of their metal intermediates discussed in the article are shown in Figure 4 and in Table 1.

## 3. Synthesis of Metal Complexes with a Fragment of Bispidine

### 3.1. Bispidine Complexes of d-Block Transition Metals

**Scheme 2:**
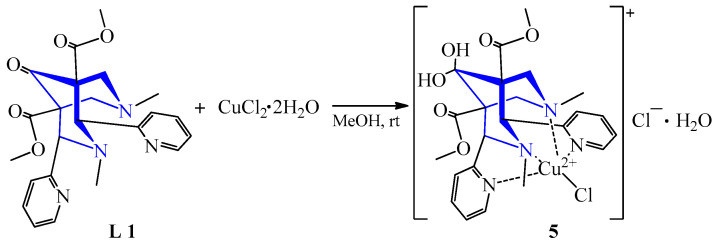
Synthesis of complex **5**.

Bispidine-type ligands (3,7-diazabicyclo[3.3.1]nonane) exhibit significant rigidity; as tetra-, penta-, and hexadentate bispidine derivatives originate from the highly rigid diazaadamantane framework. Transition metal complexes often require structural support from rigid multidentate ligands, making bispidine an ideal candidate for imparting unique coordination geometries. A representative copper-containing complex (**5**) was synthesized by stirring **L 1** in methanol (MeOH) at room temperature for 2 h with a methanolic solution of CuCl_2_·2H_2_O, followed by ether diffusion. The reaction yielded the desired complex with a 72% efficiency (Figure 4, Table 1, Scheme 2) [24].

**Scheme 3:**
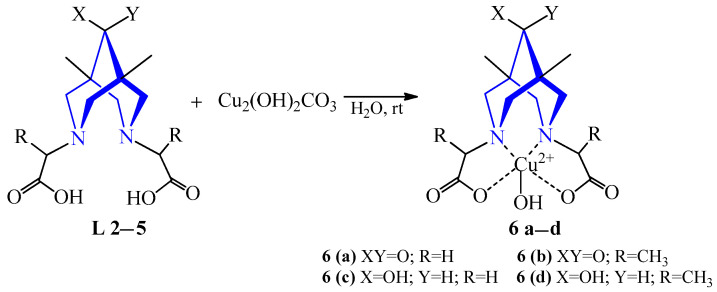
Synthesis of copper complexes **6 a**–**d**.

Solvent evaporation is commonly used to isolate target complexes following the interaction of a ligand with an inorganic salt, allowing for subsequent crystallization and solvent diffusion. However, this method may be ineffective when complexes exhibit high water solubility, preventing their formation. An alternative approach, demonstrated in [25], involves synthesizing copper complexes with an acidic N_2_O_2_-chelate. In this method, aqueous solutions of ligands **L 2**–**5** and malachite were mixed and stirred overnight, yielding copper complexes **6 a**–**d** with high efficiency (Scheme 3).

**Scheme 4:**
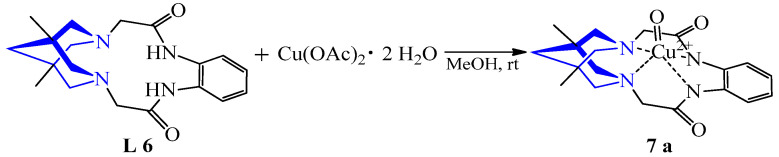
Reaction of **L 6** with copper salt.

Radioactive tracers play a crucial role in cancer diagnosis, particularly through positron emission tomography (PET). In this technique, a positron-emitting isotope binds to a biological carrier and selectively accumulates in cancerous cells. While ^62^Cu is primarily limited to perfusion studies due to its short half-life [56,57], ^64^Cu serves as an optimal positron emitter for both PET and targeted radiotherapy as, in its turn, it is an ideal [58,59,60,61]. In a related study, ligands **L 6**–**8** were combined with a Cu(OAc)_2_·2H_2_O solution and stirred overnight at room temperature, yielding copper complexes **7 a**–**c** with an efficiency of 56% (Scheme 4) [26].

**Scheme 5:**
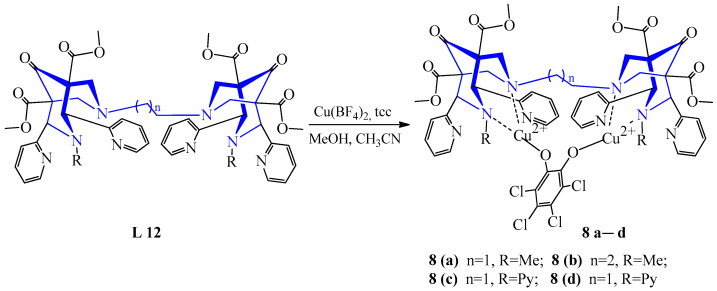
Reaction of bispidine containing ligand **L 12** with copper salt.

The oxidation of 3,5-di-tert-butylcatechin with oxygen was carried out using bispidine–copper complex catalysts **8 a**–**d** (bispidines with donor groups such as bis-tertiary amine-bispiridyl or bis-tertiary amine-trispiridyl). The catalysts **8 a**–**d**, featuring bispidine fragments, were synthesized by reacting **L 12**, 3,4,5,6-tetrachlorocatechin (tcc), and Cu(BF_4_)_2_ in the presence of acetonitrile and methanol. These reactions yielded the desired complexes with a 58% efficiency (Scheme 5) [27].

**Scheme 6:**
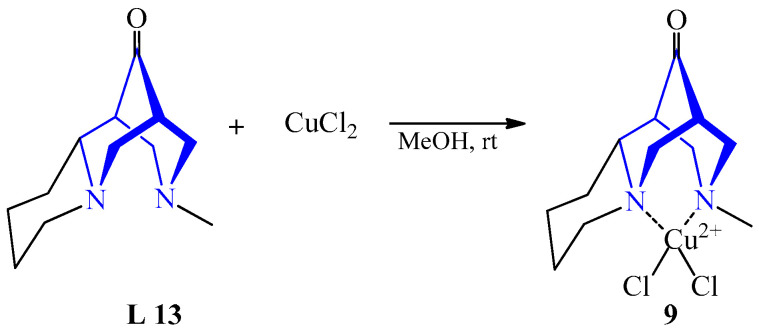
Synthesis of complex **9**.

When using bispidine ligands with copper as a catalyst in the Henry reaction, the product yield can be enhanced to 96%. To achieve this, complex **9** was synthesized reacting **L 13** with copper chloride (CuCl_2_) under continuous stirring for 5 h at 25 °C (Scheme 6) [28]. The yield of reaction products between nitromethane and 4-nitrobenzaldehyde in the presence of a copper complex **9** (20 mol % of each) reaches to 95%, and when using keratin as a catalyst, only 66% [62].

Oligonuclear transition metal complexes are of significant interest in photochemistry due to their ability to facilitate photoinduced energy and electron transfer. Ru(II) polypyridyl complexes serve as effective photoactive components, exhibiting favorable photophysical properties, such as a long excited-state lifetimes and high quantum luminescence yields. However, their application is limited by the high resistance of Ru(II) complexes to photodegradation. To address this issue, an intermediate copper complex **10**, based on the rigid bispidine ligand **L 14**, has been introduced as a potential alternative [29].

**Scheme 7:**
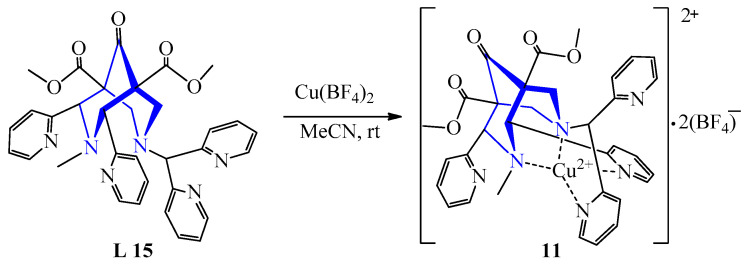
Synthesis of copper complex **11**.

Comba and colleagues (Scheme 7) designed novel ligands **L 15** and **L 16**, 3-(2-methylpyridyl)-7-(bis-2-methylpyridyl)-3,7-diazabicyclo[3.3.1]nonane, featuring two tertiary amine and four pyridine donor groups. These ligands can form both heteronuclear and mononuclear metal complexes and coordinate in different modes acting as pentadentate, hexadentate, or monodentate ligands. This versatility enables the formation of seven-coordinate pentagonal pyramidal and bipyramidal structures. The reaction of **L 15** with Cu(BF_4_)_2_ in acetonitrile yields copper complex **11** with an efficiency of 74% [30].

**Scheme 8:**
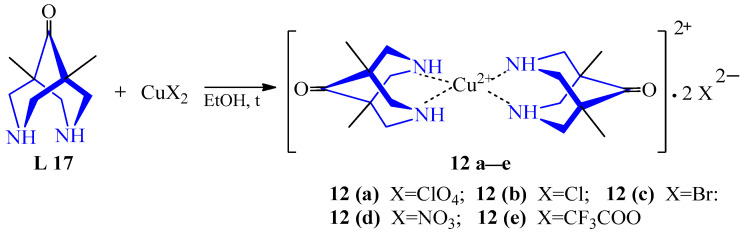
Reaction of **L 17** with copper salts.

Supramolecular metallogels (SMG) are utilized in film production, nanowire synthesis, and the removal of organic contaminants. To facilitate SMG isolation, research has focused on synthesizing bispidine ligands and their complexes, which can form coordination polymers. In this context, complexes **12 a**–**e** were synthesized with yields ranging from 51% to 95% by reacting **L 17** and a hydrate copper salt in ethanol, followed by boiling for 2 h (Scheme 8) [31].

The aziridination reaction catalyzed by transition metals holds significant importance in organic synthesis, particularly in the dihydroxylation and epoxidation of nonfunctionalized alkenes. A highly promising approach involves the transfer of nitrenes to olefins in the presence of transition metal catalysts. In [32], the synthesis of copper complexes **13 a**–**c**, based on tetra- and pentadentate bispidine ligands **L 1**, **L 18**, **L 19**, is reported for use as catalysts.

**Scheme 9:**
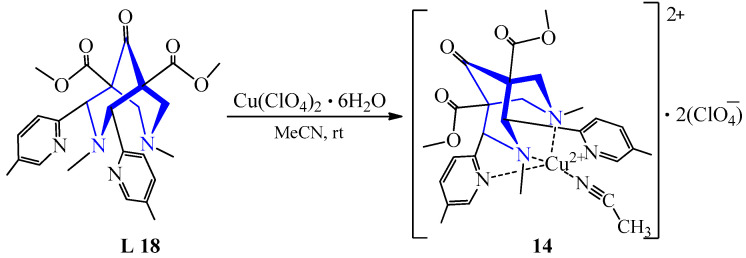
Synthesis of copper-based complex **14**.

The redox potentials and stability constants of Cu(II) complexes derived from tetradentate bispidine ligands can be changed by substituting pyridine rings. To achieve this, a suspension of **L 18** and Cu(ClO_4_)_2_·6H_2_O in acetonitrile was stirred for 2 h at room temperature, followed by ether diffusion, resulting in the isolation of complex **14** (Scheme 9) [63].

**Scheme 10:**
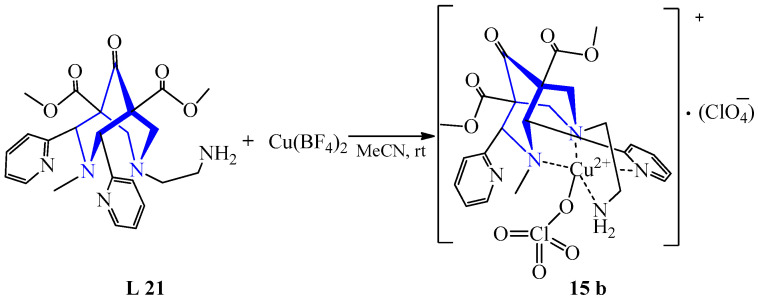
Synthesis of complex **15 b**.

Pentadentate bispidine ligands **L 20**–**22** (3,7-diazabicyclo[3.3.1]nonanes) have been demonstrated to enhance complex stability and readily interact with biological molecules and fluorescent particles for PET imaging. Additionally, these ligands rapidly form stable complexes with Cu(II). In a representative synthesis, a reaction mixture of **L 21** and copper(II) perchlorate hexahydrate in acetonitrile was stirred overnight at room temperature, followed by ether diffusion, yielding complex **15 b** as blue crystals with a 69.4% yield (Scheme 10) [33].

**Scheme 11:**
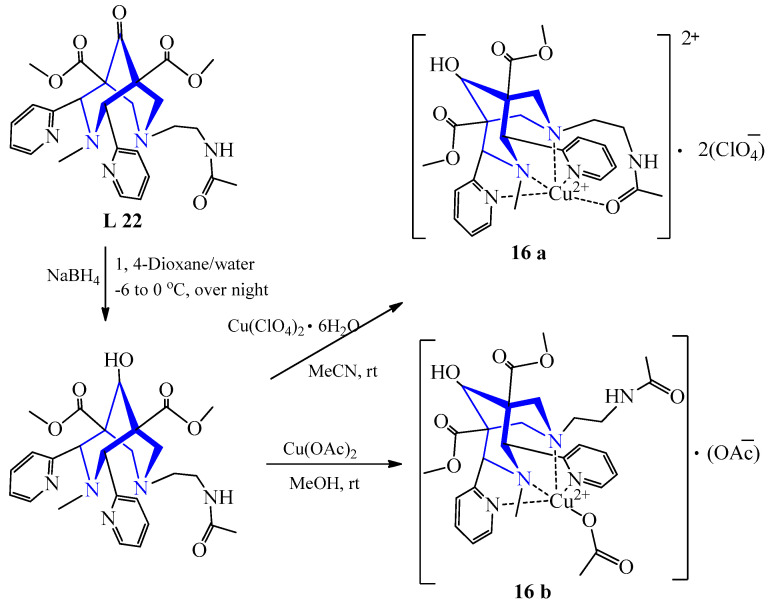
Synthesis of hexacoordinate Cu complexes **16 a**, **b**.

Bispidine ligands have demonstrated excellent efficiency in forming coordination conjugates with dyes, making them valuable for optical sensor development and imaging systems. Complexes **16 a**, **b** were synthesized from a ligand **L 22** derivative, reduced with sodium borohydride, and reacted with copper(II) acetate hexahydrate in a mixture of methanol and acetonitrile (Scheme 11) [64].

**Scheme 12:**
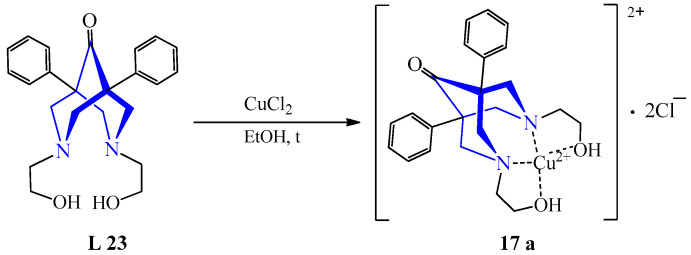
Reaction tetradentate bispidine ligand **L 23** with copper salt.

Nitrogen atoms within the rigid bicyclic framework contribute to the selectivity and stability of complex formation with metal cations and even neutral atoms [65,66,67]. Vatsadze et al. [34] synthesized metal complexes **17 a**–**d** by reacting copper(II) chloride and perchlorate with **L 23**–**26** in appropriate solvents, followed by heating the mixtures and subsequently crystallizing the products (Scheme 12).

**Scheme 13:**
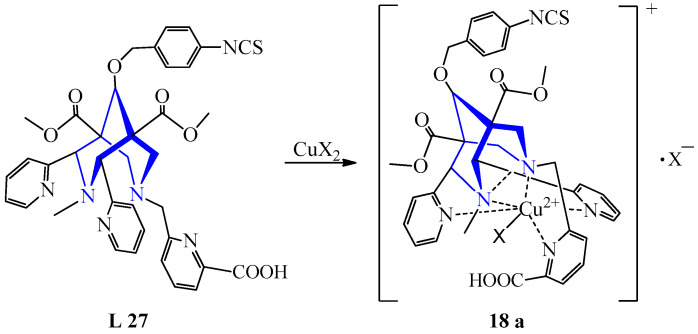
Synthesis of copper complex **18 a**.

The remarkable stability of radioactive copper complexes with bispidine-picolinates offers significant potential not only for PET imaging but also for radionuclide therapy using the therapeutic isotope copper-67 [68,69,70]. Radiopharmaceuticals based on Cu(II) ions have been synthesized through the formation of copper-containing compounds **18 a**, **b**, facilitated by the strong complexing properties of hexadentate picolinate-based bispidine ligand **L 27** (Scheme 13) [35].

**Scheme 14:**
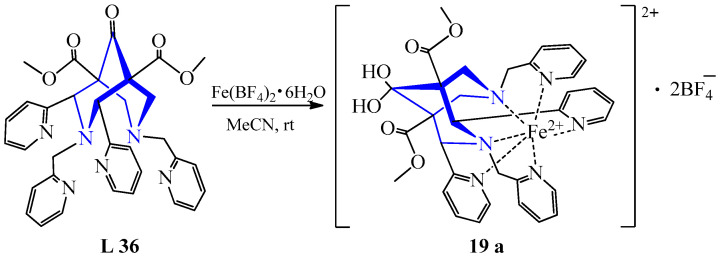
Synthesis of complex **19 a**.

Bispidinone Cu(II) complexes have been extensively studied for PET imaging due to their exceptional stability and ease of bioconjugation [71]. In addition, bispidinone-based metal chelates have been explored as catalysts for various reactions, including aziridination [72], olefin oxidation [73,74], hydroxylation of CH groups [75], halogenation [76], sulfoxidation [77], and their role in non-heme enzyme models [78,79]. Mononuclear Fe(II) coordination complexes, which exhibit low-spin, high-spin, or spin-crossover states, have found applications in biomimetic studies and magnetic resonance imaging (MRI) [80,81,82]. The reaction of **L 28**–**30** and **L 36** with iron(II) tetrafluoroborate hexahydrate or iron(II) perchlorate hydrate in degassed anhydrous acetonitrile underan argon atmosphere leads to the formation of metal complexes **19 a**–**d** with yields ranging from 17% to 71%. These complexes hold the potential to design responsive off–on probes (Scheme 14) [36].

**Scheme 15:**
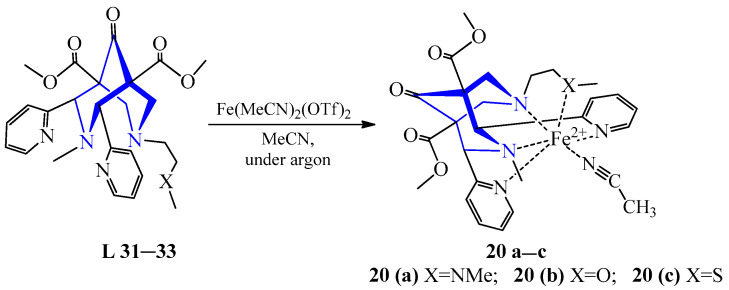
Reaction of **L 31**–**33** with an iron salt.

Iron-containing metalloproteins are well known for their essential role in mediating oxidative transformations within the human body [83,84,85]. To investigate the influence of equatorial heteroatom substitution on chlorite oxidation, Sahoo et al. [37] synthesized iron-containing complexes **20 a**–**c** by reacting **L 31**–**33** with Fe(MeCN)_2_(OTf)_2_ in acetonitrile (MeCN) at room temperature, followed by slow vapor diffusion of diethyl ether, yielding 84–88%. The complexes demonstrated the ability to oxidize ClO_2_^−^ to ClO_2_ under ambient conditions at pH 5.0 in an acetate buffer solution (Scheme 15).

**Scheme 16:**
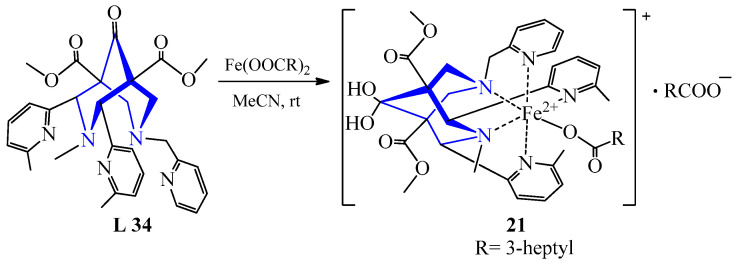
Synthesis of heptacoordinate Fe complex **21**.

High-valent iron complexes serve as catalysts in oxidation and halogenation reactions, while low-valent complexes are widely utilized in autooxidation processes within the paint industry. In a representative synthesis, iron (II) 2-ethylhexanoate was reacted with **L 34** in acetonitrile at room temperature. Following vacuum evaporation, product **21** was isolated with a 57% yield (Scheme 16) [38].

**Scheme 17:**
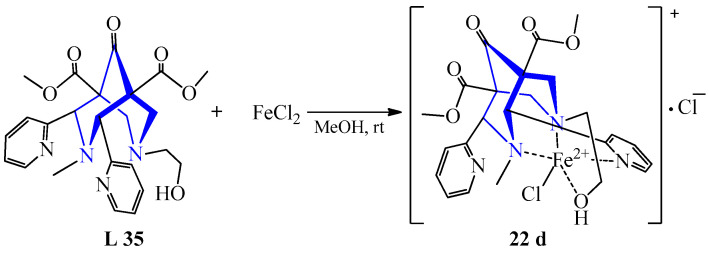
Reaction tetradentate bispidine ligand **L 35** with an iron salt.

The coordination chemistry of iron is remarkable due to its wide range of oxidation states, which play a crucial role in spin crossover phenomena and the modeling of electron and oxygen transfer enzymes. Iron(II) complexes **22 a**–**f** were synthesized using bispidine ligands **L1**, **L34**–**37**, containing two tertiary amines and two, three, or four additional donor groups (pyridine, phenolate, or alcoholate), in reaction with iron(II) salts. The resulting complexes were obtained with yields ranging from 40% to 70% (Scheme 17) [39].

**Scheme 18:**
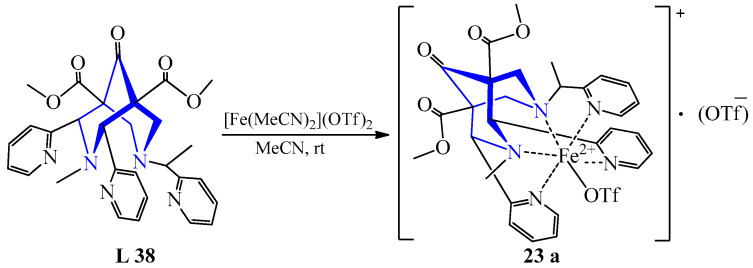
Synthesis of iron-based complex **23a**.

Certain bispidine iron–oxo complexes served as catalysts for the oxidation of non-heme iron [86,87]. Currently, several synthetic methods have been developed for such compounds, including the preparation of iron(II) complexes **23 a**, **b**. In a representative synthesis, **L 38** was reacted with iron(II) triflate in acetonitrile, yielding iron complex **23a** with a yield of 73% (Scheme 18) [40].

**Scheme 19:**
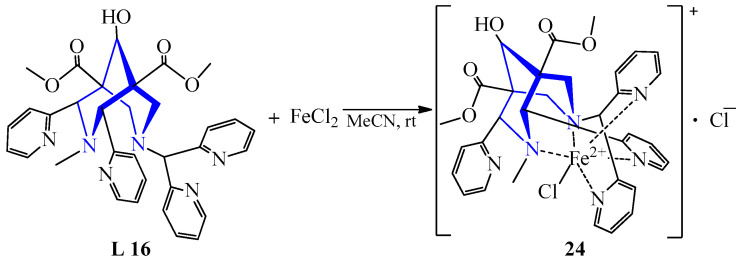
Synthesis of complex **24**.

A reaction mixture of dimethyl 7-(di(pyridine-2-yl)methyl)-9-hydroxy-3-methyl-2,4-di(pyridine-2-yl)-3,7-diazabicyclo[3.3.1]nonan-1,5-dicarboxylate **L 16** and ferric chloride (FeCl_2_) in dry acetonitrile was stirred at room temperature overnight. The resulting yellow solution was subjected to diethyl ether diffusion, yielding yellow needle-like crystals of complex **24** with a 52% yield (Scheme 19) [30].

**Scheme 20:**
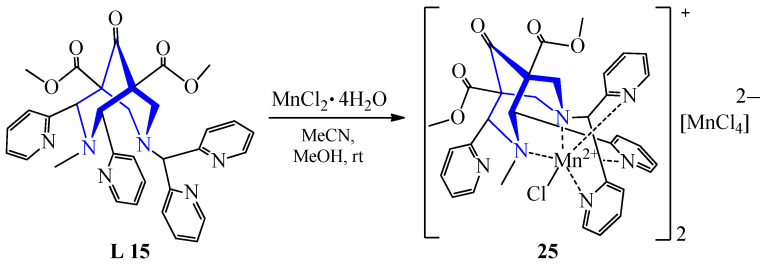
Reaction of **L 15** with manganese salt.

Bispidine Mn(II) complexes with tetradentate ligands typically adopt an octahedral geometry, whereas heptacoordinated Mn(II) complexes can exhibit pentagonal bipyramidal or trigonal prismatic structures. In a representative synthesis, **L 15** and MnCl_2_·4H_2_O were suspended in an equimolar acetonitrile–methanol mixture and stirred overnight at room temperature, followed by diethyl ether diffusion. The reaction yielded complex **25** with an efficiency of 56% (Scheme 20) [30].

**Scheme 21:**
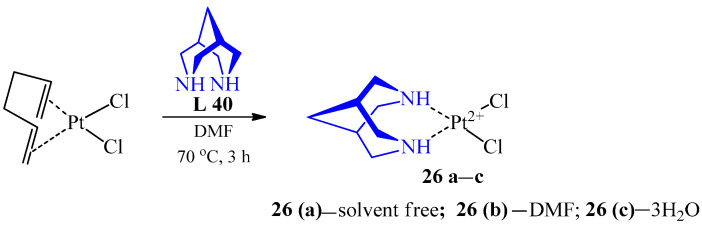
Reaction of **L 40** with a platinum salt.

The platinum complex cisplatin (cis-(NH_3_)_2_PtCl_2_) exhibits significant pharmacological properties, including alkylating, immunosuppressive, antitumor, and cytostatic effects. This has driven extensive research into the properties of platinum complexes and the development of efficient synthetic methods. In the pursuit of Pt complexes with cytotoxic activity, Cui et al. [41] synthesized platinum complexes **26 a**–**c** by reacting (1,5-hexadiene)PtCl_2_ with bispidine **L 40** in dimethylformamide (DMF) at 70 °C for 3 h (Scheme 21).

**Scheme 22:**
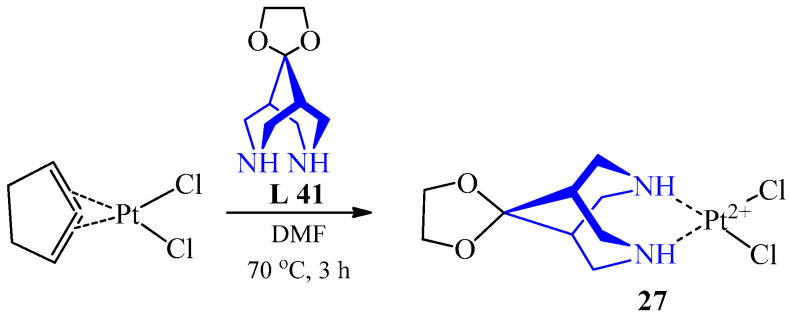
Synthesis of tetracoordinate Pt complex **27**.

Researchers [42] synthesized (spiro[bispidin-9.2′-[1,3]dioxolan])platinum (II) dichloride (**27**) by reacting **L 41** with (C_6_H_10_)PtCl_2_ in dimethylformamide (DMF). The reaction was conducted at 100 °C for 2 h, yielding the product with an efficiency of 97% (Scheme 22).

**Scheme 23:**
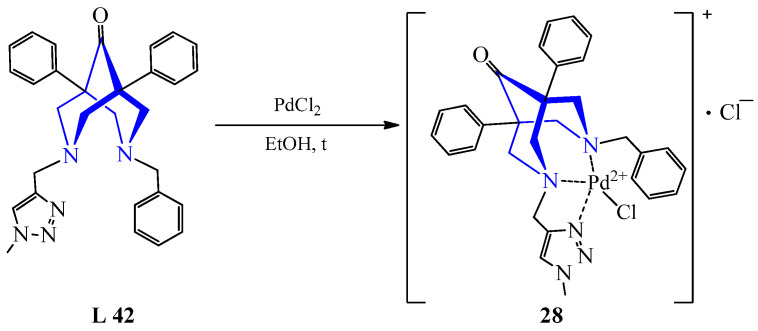
Reaction of **L 42** with palladium salt.

Pyrazole rings, macrocyclic amides, and oxygen-containing substituents (such as alcohols or carboxylate groups) can enhance the number of coordination sites within the bispidine core [88]. To expand its coordination capacity, a, palladium-containing complex (**28**) was synthesized by reacting PdCl_2_ with **L 42**, which was obtained via the CuAAC click reaction, in acetonitrile at room temperature for 48 h (Scheme 23) [43].

**Scheme 24:**
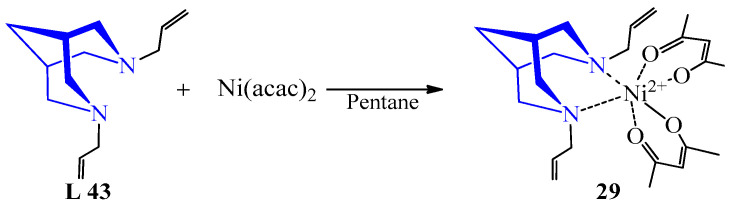
Synthesis of nickel complex **29**.

The ligand **L 43** has been found to be a highly organized bidentate ligand, with its alkyl substituents providing functional groups for further modifications. Nickel acetylacetonate Ni(acac)_2_ was reacted with 3,7-Diallyl-3,7-diazabicyclo[3.3.1]nonane **L 43** in the presence of pentane for 1 h at room temperature, yielding (C_7_H_12_N_2_allyl_2_)Ni(acac)_2_ **29** with an efficiency of 99% (Scheme 24) [44].

**Scheme 25:**
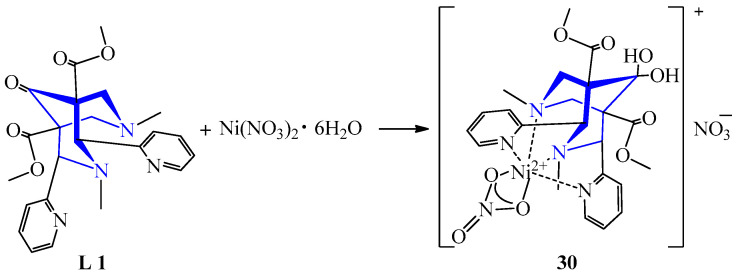
Synthesis of complex **30**.

The formation of C(sp^2^)–C(sp^3^) bonds is a fundamental transformation in organic synthesis. It has been established that sp, sp^2^, and sp^3^ carbon atoms can be effectively linked using nickel catalysts. The (bispidine)Ni(NO_3_)_2_ complex **30**, which exhibits strong reactivity towards various aryl halides with benzylzine bromides or dialkylzine reagents, was synthesized with a 98% yield by reacting **L 1** with nickel nitrate hydrate (Scheme 25) [45].

**Scheme 26:**
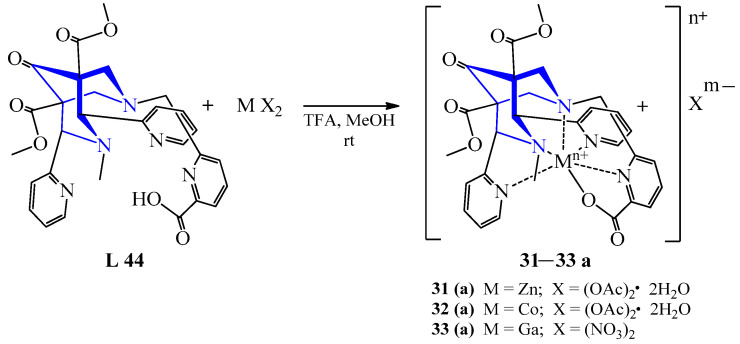
Reaction of **L 44** with various metal salts.

The stability of metal complexes under physiological conditions is a critical factor in nuclear medicine, which can be achieved using rigid ligands with metal-binding capabilities. The coordination chemistry of hexadentate ligands **L 44**–**45** with various metals ions (Zn(II), Co(II), and Ga(III)) was investigated. The reaction of **L 44** and **L 45** with metal salts resulted in the formation of complexes **31**–**33** with yields ranging from 32 to 98% (Scheme 26) [46].

**Scheme 27:**
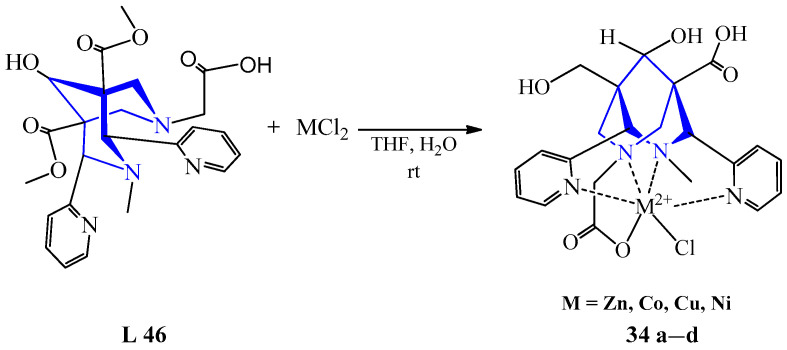
Reaction of **L 46** with metal salts.

Positron emission tomography (PET) combined with X-ray computed tomography (CT) provides highly accurate information on tumor progression in various cancers, including lymphoma and epithelial malignancies affecting the lungs, esophagus, cervix, head, and neck [89,90,91]. However, a key limitation arises from the slow redistribution kinetics of bioconjugates, which may not always align with non-metallic isotopes. To overcome this challenge, the development of metal radioisotopes, specifically metal complexes **34 a**–**d**, with longer half-lives and simplified radiochemistry, is essential [92]. In pursuit of such compounds, a recent study synthesized coordination complexes of Zn(II), Co(II), Cu(II), and Ni(II) with **L 46** (Scheme 27) [47,48].

**Scheme 28:**
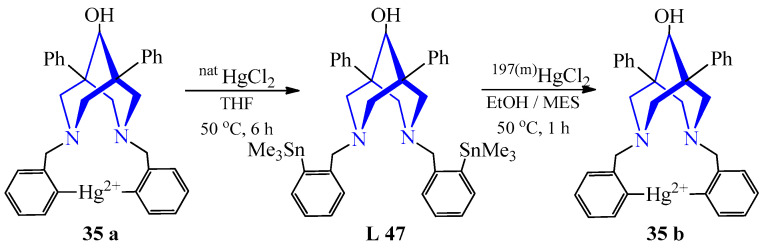
Synthesis of mercury complexes **35 a**, **b**.

Mercury-197 (^197^Hg) complexes have been clinically utilized in diagnostics imaging of the kidneys and brain, particularly in the form of chloroperodrine with a radioactive label, due to their stability and the radiochemical properties used. Mercury complexes with a bispidine framework (**35 a**, **b**) demonstrated high chemical stability in the presence of an excess of sulfur-containing compounds and exhibited strong in vivo stability (Scheme 28) [49].

### 3.2. Complexes of p-Block Elements with Bispidines

**Scheme 29:**
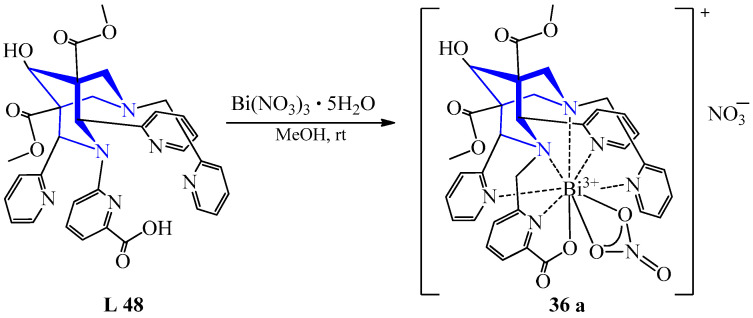
Synthesis of bismuth-based complex **36 a**.

There is a significant demand for bismuth-based complexes, driven by the demonstrated effectiveness of targeted cancer radiotherapy using actinium-225, whose daughter isotopes (bismuth-211 and bismuth-213) exhibit high therapeutic potential. The synthesis of complexes **36 a**–**e** was achieved by reacting bispidine ligands **L48**–**51** with bismuth nitrate hydrate in the presence of methanol, yielding 60% to 99% (Scheme 29) [50].

**Scheme 30:**
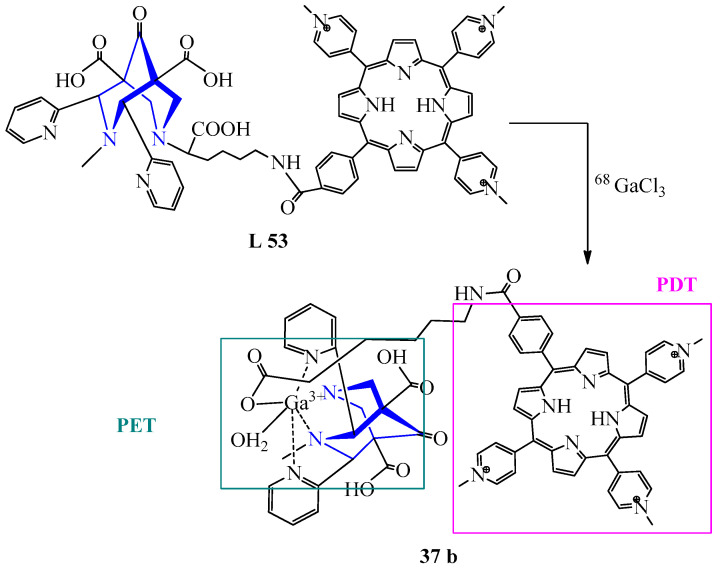
Synthesis of complex **37 b**.

Gallium-68 (^68^Ga) complexes are widely utilized in PET/PDT for early disease detection, allowing diagnosis before physical symptoms manifest, as gallium nuclides facilitate efficient targeting of disease sites. Complexes **37 a**, **b** were synthesized with a 90% yield by reacting ligands **L 52** and **L 53** with GaCl_3_ in aqueous suspension overnight at 100 °C (Scheme 30) [51].

**Scheme 31:**
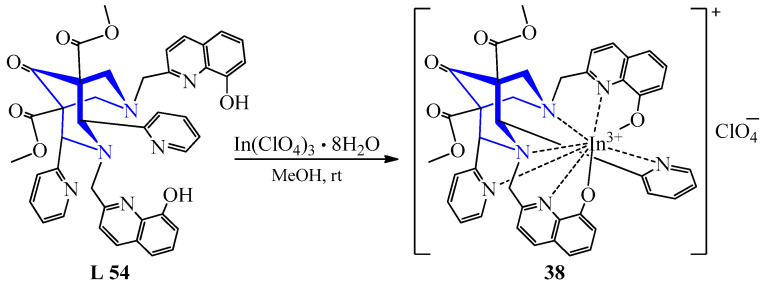
Reaction heptadentate bispidine ligand **L 54** with an indium salt.

Since the first clinical studies in 1925 demonstrated the effectiveness of bismuth-214 complexes for measuring blood flow between hands, scientific efforts have focused on developing new methods for utilizing other complexes in single-photon emission computed tomography (SPECT), positron emission tomography (PET), and targeted therapies (including alpha, beta, and electron therapy). In a representative synthesis, indium complex **38** was obtained with a 39% yield by reacting bispidine ligand **L 54** with indium perchlorate hydrate In(ClO_4_)_3_·8H_2_O at room temperature for 5 h (Scheme 31) [52].

### 3.3. Bispidine-Containing Lanthanide Complexes

**Scheme 32:**
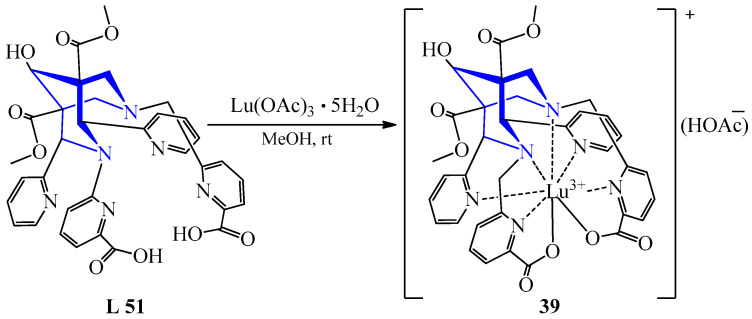
Reaction hexadentate bispidine ligand **L 51** with a lutetium salt.

The reaction of **L 51** with indium, lanthanum and lutetium salts resulted in the formation of complexes **39**–**41**, with yields ranging from 81% to 88% (Scheme 32) [53].

**Scheme 33:**
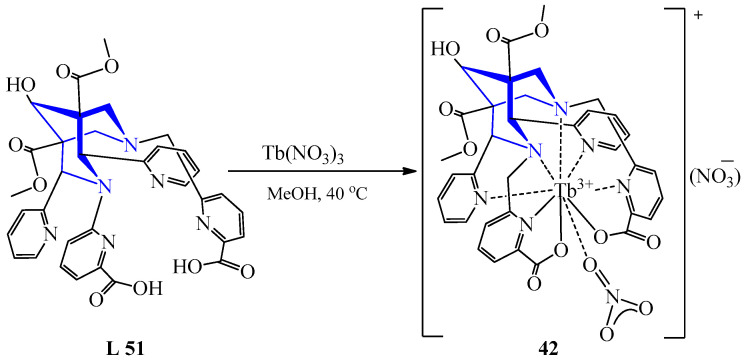
Synthesis of terbium complex **42**.

The bispidine ligand **L 51**, functioning as an antenna, effectively transferred energy to the lanthanides metal centers, which exhibited high radiation intensity and extended luminescence lifetimes. This highlights the potential of complexes **42**–**44** for use in fluorescent probe fabrication. A notable example is the terbium complex **42**, synthesized through the reaction of **L 51** with terbium nitrate (Scheme 33) [54].

**Scheme 34:**
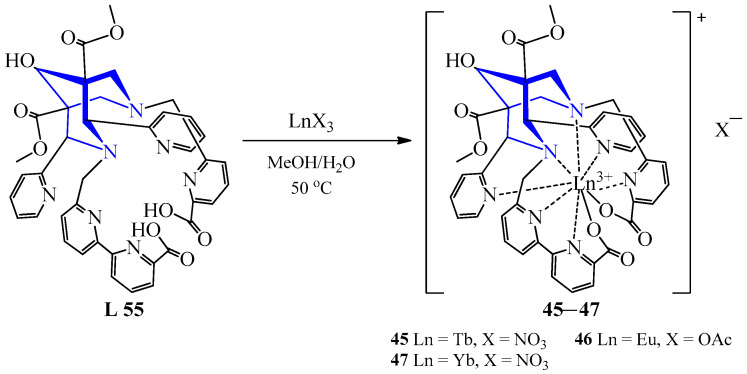
Synthesis of complexes **45**–**47**.

Lanthanide complexes **45**–**47**, featuring a bispidine framework with photophysical properties, were synthesized using readily available materials, including **L 55** and methanol in an aqueous solutions of lanthanide salts. The resulting products were obtained with yields ranging from 40% to 70% (Scheme 34) [55].

The rigid framework and tunable basicity of bispidine ligands significantly influence the stability and selectivity of their metal complexes, enabling the synthesis of tetra-, penta-, hexa- and octadentate metal complexes incorporating a bispidine fragment. These complexes have been applied in various catalytic and biomedical fields. In organic synthesis, bispidine-based copper(II) complexes catalyze aziridination reactions [25,93,94], while nickel (II) pre-catalyst facilitate C-C bond formation [95]. Additionally, copper(II) complexes play a role in the enantioselective Henry reaction [96,97,98]. Other notable applications include oxygen activation using copper (I) and cobalt (II) [99,100] and oxidation reactions mediated by high-valent iron and manganese complexes with bispidine ligands [101,102]. In medicine, bispidine complexes are explored as potential cytostatic agents in cancer therapy [103]. In radiopharmaceuticals, they are widely used for PET/PDT imaging [104,105].

## 4. Conclusions

Bispidines constitute a diverse class of ligands well-established in coordination chemistry, exhibiting coordination numbers ranging from 4 to 8. Their adaptability in terms of coordination geometry, donor group variability, and structural rigidity allows for the tailored design of bispidine ligands for specific metal ions and diverse applications.

In radiopharmaceutical development, key factors include efficient radiolabeling, complex stability, and the ease of functionalization for conjugation with biological vectors. This review highlights recent advances in the chemistry of bispidine–metal complexes, demonstrating their high efficiency and broad practical applicability. These developments underscore the significant potential of bispidine complexes in medical and radiopharmaceutical chemistry, particularly for drug development and catalytic systems that mimic natural enzymes. Furthermore, ongoing research in coordination chemistry is expected to yield new discoveries, expanding the scope of bispidine applications.

## Figures and Tables

**Table 1:** Some characteristics of various metal complexes containing bispidine framework.

No	Salts	Lig. (L)	Complex, Solvent	λ_max_ {ε(M^−1^ cm^−1^)}	Yield, %	The Color of the Solid	Synthesis Method	Application	Ref.
1	CuCl_2_·2H_2_O	L 1	[Cu(L)Cl]Cl·2H_2_O, MeOH	656 {90}	72	Blue	Diffusion of ether		[24]
2	Cu_2_(OH)_2_CO_3_	L 2–5	[L_2_Cu(OH)_2_], H_2_O	602–612 {247–252}		Blue	Neutralization with acidic chelate N_2_O_2_	Radiopharmaceuticals, positron emission tomography (PET)	[25]
3	Cu(OAc)_2_·2H_2_O	L 6 L 7–8	[Cu(L)(O)], MeOH [Cu(L)(OH_2_)], MeOH	483 {149} 476 {117} 524 {189}	56	Red and purple	Recrystallization	^64^Cu PET visualization	[26]
4	Cu(BF_4_)_2_	L 1, L 9–12	[Cu(L)NCMe)]^2+^, MeCN [Cu_2_(L)NCMe)]^4+^, MeCN, MeOH	450 {510} 453 {577} 456 {685} 468 {651} 532 {1640}	58			Catalytic enzymatic oxidation of catecholamines	[27]
5	CuCl_2_	L 13	[Cu(L)Cl]Cl, MeOH		55	Green	Recrystallization	The catalyst for Henry’s enantioselective reaction	[28]
6	Cu(ClO_4_)_2_·6H_2_O	L 14	[Cu(L)](ClO_4_)_2_, MeOH:H_2_O	412	70	Green	Heating, concentration at low pressure	The aziridination reaction	[29]
7	Cu(BF_4_)_2_	L 15 L 16	[Cu(L)](BF_4_)_2_·3H_2_O, MeCN [Cu(L)](BF_4_)_2_·2H_2_O, MeCN	626 {119} 630 {112}	74 54	Blue	Evaporation, diffusion of ether heating, diffusion of ether	The aziridination reaction	[30]
8	Cu(ClO_4_)_2_·6H_2_O CuCl_2_·2H_2_O CuBr_2_·2H_2_O Cu(NO_3_)_2_·3H_2_O Cu(CF_3_COO)_2_·4H_2_O	L 17	[Cu(L)_2_](ClO_4_)_2_, EtOH [Cu(L)_2_Cl]Cl, EtOH [Cu(L)_2_Br]Br, EtOH [Cu(L)_2_](NO_3_)_2_, EtOH [Cu(L)_2_](CF_3_COO)_2_, EtOH	284 {450} 273 {462} 272 {482} 288 {450} 284 {485}	95 - 68 51 -	Pink Blue Purple Yellow	Concentration at low pressure, recrystallization	Development of supramolecular coordination polymers	[31]
9	Cu(CF_3_SO_3_)_2_ Cu(BF_4_)_2_·6H_2_O	L 1 L 18 L 19	[Cu(L)](CF_3_SO_3_)_2_, MeCN [Cu(L)](BF_4_)_2_·3H_2_O, MeCN [Cu(L)](BF_4_)_2_·4H_2_O, MeCN	- 624 {25} 881 {60}	83 88 89		Diffusion of ether	The aziridination reaction	[32]
10	Cu(ClO_4_)_2_·6H_2_O	L 20–21 L 22	[Cu(L)](ClO_4_)_2_, MeCN [Cu(L)](ClO_4_), MeCN	629 627 564	84 69 92	Blue Blue Purple	Diffusion of ether	^64^Cu PET visualization	[33]
11	CuCl_2_ Cu(ClO_4_)_2_ Cu(OH)_2_	L 23 L 24 L 25 L 26	[Cu(L)]Cl_2_, EtOH [Cu(L)](ClO_4_)_2_, CHCl_3_ [Cu(L)](OH)_2_, DMF			Blue Purple Dark- blue	Heating, recrystallization	Catalysts for enantioselective reactions	[34]
12	CuX_2_	L 27	[Cu(L)X]X					For the synthesis of radiopharmaceuticals based on Cu(II) ions	[35]
13	Fe(BF_4_)_2_·6H_2_O Fe(ClO_4_)_2_·H_2_O Fe(BF_4_)_2_·6H_2_O	L 36 L 28 L 29 L 30	[Fe(L)] 2BF_4_, MeCN [Fe(L)]·2ClO_4_, MeCN [Fe(L)(MeCN)]·2BF_4_, MeCN	458 {5560} 455 {10,870} 443 {12,570} 444 {7144}	71 36 25 17	Red-black Dark-brown - Dark-brown	Diethyl ether diffusion, heating, recrystallization	For the design of responsive off–on probes	[36]
14	Fe(MeCN)_2_(OTf)_2_	L 31 L 32 L 33	[Fe(L) MeCN)]^2+^, MeCN	37 {1061] 380 {1057} 373 {1112}	88 84 88	Yellow Brown Light-yellow	Slow vapor diffusion with diethyl ether	For oxidizing ClO_2_^−^ to ClO_2_	[37]
15	Fe(OOCC_7_H_15_)_2_	L 34	[Fe(L)OOCC_7_H_15_] (OOCC_7_H_15_)	400	57	Canary	Evaporation, recrystallization	Catalyst for the auto-oxidation process	[38]
16	[Fe(NCCH_3_)_2_·(CF_3_SO_3_)_2_] Fe(SCN)_2_ FeSO_4_ FeCl_2_ Fe(ClO_4_)_2_ FeCl_2_	L 1 L 1 L 34 L 35 L 36 L 37	[Fe(L)(CF_3_SO_3_)_2_], CD_3_CN [Fe(L)(SCN)_2_], CD_3_CN [Fe(L)SO_4_], MeOH [Fe(L)Cl]Cl, MeOH [Fe(L)](ClO_4_)_2_, MeOH [Fe(L)Cl]Cl, MeOH	392 375 404 412 {1819} 564 {215} 579 {948}	40–70		Ether diffusion, recrystallization	Catalysts	[39]
17	[Fe(NCCH_3_)_2_]·(CF_3_SO_3_)_2_	L 38 L 39	[Fe(L)(CF_3_SO_3_)] (CF_3_SO_3_), MeCN [Fe(L)(CF_3_SO_3_)] (CF_3_SO_3_)·3H_2_O, MeCN		73 33	Yellow	Recrystallization	A catalyst for bioinspired catalysis	[40]
18	FeCl_2_ Fe(ClO_4_)_2_ Fe(BF_4_)_2_·6H_2_O	L 16 L 15 L 15	[Fe(L)Cl]Cl, MeCN [Fe(L)(OCH_3_)](ClO_4_)_2_, MeOH [Fe(L)(OCH_3_)](BF_4_)_2_, MeOH	412 {1381} - 364 {1245}	52 49 54	Yellow	Diffusion of ether	The activation process	[30]
19	MnCl_2_·4H_2_O	L 15	[Mn(L)Cl]_2_[MnCl_4_], MeCN: MeOH		56	Colorless	Evaporation, diffusion of ether	PET visualization	[30]
20	(C_6_H_10_)PtCl_2_	L 40	[Pt(L)Cl]Cl, DMF [Pt(L)Cl]Cl·C_3_H_7_NO, DMF [Pt(L)Cl]Cl·3H_2_O, H_2_O		87 87 60	Yellow	Recrystallization, cooling heating, recrystallization	Cytotoxicity against human cancer cell lines K562 (chronic myeloid leukemia), A2780 (ovarian cancer)	[41]
21	(C_6_H_10_)PtCl_2_	L 41	[Pt(L)Cl]Cl, DMF		97	Yellow	Heating, recrystallization	Preparation of compounds with cytotoxic activity	[42]
22	PdCl_2_	L 42	[Pd(L)Cl]Cl, MeCN					Might be used as catalysts under microwave irradiation	[43]
23	Ni(acac)_2_	L 43	[Ni(L)](acac)_2_, C_5_H_12_		99	Blue	Cooling	Immobilization of metal heterogeneous surfaces	[44]
24	Ni(NO_3_)_2_·6H_2_O	L 1	[Ni(L)(NO_3_)](NO_3_), MeCN: MeOH		98	Pink	Evaporation, recrystallization	A catalyst for bond formation C(sp^2^)-C(sp^3^)	[45]
25	Zn(OAc)_2_·2H_2_O	L 44 L 45	[Zn(L)](TFA), TFA, MeOH		98 61	Colorless	Evaporation, diffusion of ether	Bifunctional Chelators for ^64^Cu PET	[46]
26	ZnCl_2_	L 46	[Zn(L)Cl], THF, H_2_O				Evaporation, centrifugation	Chelator for radiolabeling studies with 64Cu	[47,48]
27	Co(OAc)_2_·2H_2_O	L 44 L 45	[Co(L)](TFA), TFA, MeOH	470 {123} 475 {117}	32 67	Red Brown	Evaporation, diffusion of ether	Bifunctional Chelators for ^64^Cu PET	[46]
28	^197^HgCl_2_ ^Nat^HgCl_2_	L 47	[Hg(L)], EtOH [Hg(L)], THF				Heating, evaporation	Theranostic applications in radiopharmaceuticals	[49]
29	Bi(NO_3_)_3_·5H_2_O	L 48 L 49 L 50 L 51	[Bi(L)](NO_3_)_2_, MeOH [Bi(L)(NO_3_)](NO_3_), MeOH [Bi(L)(Br)_0,5_(NO_3_)_0,5_], MeOH [Bi(L)(NO_3_)], MeOH [Bi(L)(NO_3_)](NO_3_), MeOH		93 99 60 95 67		Recrystallization Evaporation, recrystallization	Targeted ^213^Bi alpha therapy	[50]
30	GaCl_3_	L 52 L 53	[Ga(L)]Cl, H_2_O	- 422 {240}	90	White Purple	Heating, recrystallization	PET visualization, creating stable theranostic probes for PET/PDT	[51]
31	Ga(NO_3_)_3_	L 48	[Ga(L)](TFA)(NO_3_), TFA, MeOH:H_2_O		65	Colorless	Heating, evaporation	Bifunctional Chelators for ^64^Cu PET	[46]
32	In(ClO_4_)_3_·8H_2_O	L 54	[In(L)](ClO_4_), MeOH		39	Orange	Concentration in a vacuum	Creation of a chelating component in radiopharmaceuticals based on In	[52]
33	In(OAc)_3_·5H_2_O Lu(OAc)_3_·4H_2_O LaCl_3_·7H_2_O	L 51	[In(L)](TFA), TFA, MeOH, H_2_O [Lu(L)](TFA), TFA, MeOH [La(L)](TFA), TFA, MeOH		88 84 81	Colorless	Evaporation Evaporation, diffusion of ether Evaporation, concentration in vacuum	Chelators in nuclear medicine used in single-photon emission computed tomography (SPECT)	[53]
34	Nd(OAc)_3_ Tb(NO_3_)_3_ Dy(OAc)_3_	L 51	[Nd(L)(OH)_2_](TFA)·(MeOH), TFA, MeOH [Tb(L)(OH)_2_](TFA)·2H_2_O·MeCN, TFA, H_2_O, MeCN [Dy(L)(OH)_2_](TFA)·1.5H_2_O·MeCN, TFA, H_2_O, MeCN	- 491 -		Colorless	Evaporation, diffusion of ether	Acting as an antenna for energy transmission	[54]
35	Tb(NO_3_)_3_ Eu(OAc)_3_ Yb(NO_3_)_3_	L 55	[Tb(L)](NO_3_) MeOH·H_2_O, MeOH:H_2_O [Eu(L)](OAc), EtOH:MeOH [Yb(L)] (NO_3_) MeOH, H_2_O, MeOH,	261 261 262	40–70		Heating, evaporation	Biological fluorescent probes with red-shifted absorption	[55]

## Data Availability

The datasets used and/or analyzed during the present study are available from the corresponding author on reasonable request.
